# Feasibility and relevance of urine culture during stone fragmentation in patients undergoing percutaneous nephrolithotomy and retrograde intrarenal surgery: a prospective study

**DOI:** 10.1007/s00345-020-03387-6

**Published:** 2020-07-30

**Authors:** E. De Lorenzis, L. Boeri, A. Gallioli, M. Fontana, S. P. Zanetti, F. Longo, R. Colombo, M. Arghittu, S. Piconi, G. Albo, A. Trinchieri, E. Montanari

**Affiliations:** 1 Department of Urology, Fondazione IRCCS Ca’ Granda Ospedale Maggiore Policlinico, Via della Commenda 15, 20122 Milan, Italy; 2 Department of Clinical Sciences and Community Health, University of Milan, Milan, Italy; 3 Microbiology and Virology, Fondazione IRCCS Ca’ Granda Ospedale Maggiore Policlinico, Milan, Italy; 4 Infectious Diseases Department, ASST Fatebenefratelli-Sacco, Milan, Italy; 5 University of Milan, Milan, Italy

**Keywords:** Urinary tract infection, Urolithiasis, Culture media, Ureteroscopy, Percutaneous nephrolithotomy, Sepsis

## Abstract

**Purpose:**

We evaluated if, during lithotripsy, bacteria may be detected in the irrigation fluid of percutaneous nephrolithotomy (PCNL) and retrograde intrarenal surgery (RIRS). The concordance between urine culture from stone fragmentation (SFUC), bladder (BUC), renal pelvic (RPUC) and stone (SC) was analyzed. We also assessed the correlation between variables and cultures and their association with systemic inflammatory response syndrome (SIRS) and of a positive SC.

**Methods:**

We included 107 patients who underwent PCNL (*n* = 53) and RIRS (*n* = 54) from January 2017 to May 2018. Samples for RPUC were obtained by renal catheterization. Stone fragments and irrigation fluid sample were sent for culture.

**Results:**

SFUC was positive in 17 (15.9%), BUC in 22 (20.6%), RPUC in 26 (24.3%) and SC in 30 patients (28%). The concordance between SFUC and SC was the highest among all cultures: 94.1%. SFUC and SC grew identical microorganisms in 15/17 (88.2%) patients. Out of 17 (15.9%) patients with SIRS, 8 (7.5%) had sepsis. SFUC had the highest PPV and specificity to detect positive SC and SIRS. Previous urinary tract infection, a preoperative nephrostomy, stone diameter and composition, staghorn calculi, PCNL, positive BUC, RPUC and SFUC were predictors of infected stone. Variables that indicate complex stones, complex PCNL and an infection of the upper tract were associated with SIRS.

**Conclusion:**

SFUC is technically feasible, easy to retrieve and to analyze. The spectrum of SFUC potential application in clinical practice is when is not possible to perform a SC, e.g. complete dusting or during micro-PCNL.

**Electronic supplementary material:**

The online version of this article (10.1007/s00345-020-03387-6) contains supplementary material, which is available to authorized users.

## Introduction

Infections are the most common complications of endourological procedures for stones. Among all patients submitted to percutaneous nephrolithotomy (PCNL), 10–30% develop fever, 35% systemic inflammatory response syndrome (SIRS), and 0.9–9.3% sepsis after surgery [1–3]. Using a standardized method for the definition and classification, infective complications post retrograde intrarenal surgery (RIRS) occur in 7.7% of patients, consisting of fever (4.4%), SIRS (1.7%), and sepsis (0.7%) [4].

In order to prevent infectious complications, the European Association of Urology guidelines on urolithiasis recommend a preoperative bladder culture (BUC) and perioperative antibiotic prophylaxis before endourological stone removal [5].

However, BUC is an inaccurate predictor of infections following endourology, since it does not represent the microbiological status of the upper urinary system, especially in cases of obstruction or infected stones [6]. Stone and pelvic urine cultures were found to be more accurate predictors of postoperative infections [7, 8].

Several pathogenic mechanisms underlying infectious complications after urolithiasis surgery have been postulated: the bacterial colonization of stones which might not allow for preoperative sterilization despite targeted antibiotics [9]; the release of endotoxins during lithotripsy [10, 11]; the generation of high intrarenal pressure, which contributes to pyelotubular and pyelovenous backflow and can introduce bacteria into systemic circulation [12, 13]; and the intrinsic renal vascular damage produced by the percutaneous access [14, 15].

Considering these factors, we postulated that stone-colonizing bacteria may be disseminated in the renal collecting system during lithotripsy.

We evaluated the following: (i) the detection of bacteria released during stone fragmentation in the irrigation fluid and its clinical relevance; (ii) the concordance between urine culture during stone fragmentation (SFUC), BUC, renal pelvic urine culture (RPUC), and stone culture (SC); (iii) the correlation between the analyzed variables and cultures and their association with the risk of postoperative SIRS and of a positive SC.

## Patients and methods

### Data collection

We performed a prospective study in one academic hospital including all patients ≥ 18 years who underwent PCNL and RIRS between January 2017 and May 2018 for urolithiasis.

All patients underwent a BUC 2–3 weeks preoperatively. Patients with negative BUC received a first generation cephalosporin as perioperative prophylaxis. Patients with an asymptomatic bacteriuria started a targeted therapy 48–72 h before intervention. In cases of leukocytosis, urinary symptoms or fever, or a history of urosepsis, the surgery was postponed until after a full antibiotic course and a negative BUC.

A urine sample was collected from the renal pelvis at the beginning of the procedure. After lithotripsy of approximately half of the stone burden, we stopped the irrigation flow and continued fragmentation as long as visibility remained clear. At this moment, a sample of irrigation fluid was collected through the operative channel of the instrument.

Extracted stones were sent for SC and biochemical analysis with infrared spectrophotometry.

SIRS was defined as the presence of at least 2 of the following criteria: fever (> 38 °C) or hypothermia, tachypnea (> 20 breaths per min) or PCO_2_ < 32 mmHg, tachycardia (> 90 beats per min), leukocytosis (> 12,000 cells/mm^3^), and leukopenia (< 4000 cells/mm^3^). Sepsis was considered to be SIRS associated with suspicion or confirmation of an infection caused by pathogenic or potentially pathogenic micro-organisms [16].

In case of postoperative infectious complications, the prophylactic antibiotic was continued as a full-course regimen; non-responding patients were treated with an empirical broad-spectrum antibiotic. In all cases, the therapy was targeted on intraoperative culture results when available.

Complications were analyzed according to the Clavien–Dindo classification [17].

Data collection followed the principles outlined in the Declaration of Helsinki. The ethical committee approved this study.

### Microbiology laboratory technique

BUC was tested both for bacteria and fungi. Concerning the quantitative bacterial count of the urine referring to Kass Index [18], a growth ≥ 10^5^ CFU/mL was considered as positive.

Regarding RPUC, because of the paucity of the urine collected from the renal pelvis, the sample was stored in a pediatric blood culture bottle to optimize microorganism growth. Stone fragments were collected in a sterile bottle containing 1 mL of 0.9% saline; 10 µl of the sample was inoculated in the same way as RPUC.

The sample retrieved during fragmentation (about 5 ml) was centrifuged to obtain a pellet of 1 mL, of which 10 µL was cultured on CPS ID3 (bioMérieux, Marcy-l’Étoile, France) and 10 µL on Sabouraud Dextrose Agar + CAF (bioMérieux), as for BUC.

Considering that Kass Index may be a weak criterion to evaluate the positivity of samples other than midstream urine, referring to RPUC, SFUC, and SC, growths ≥ 10^3^ CFU/mL were considered as positive. The isolated strain, monomicrobic positivity and time to detection were used to discriminate possible contamination.

In case of fever, two sets of blood samples were cultured on standard aerobic and anaerobic culture.

Antimicrobial susceptibility tests (AST) were performed in accordance with European Committee on Antimicrobial Susceptibility Testing criteria [19].

### Surgical technique

#### Retrograde intrarenal surgery

After a rigid ureteroscopy, a ureteral access sheath (UAS) was placed (10–12 or 11–13 ch). In all patients, fragments were removed using baskets. A re-usable flexible scope was employed. A DJ stent or a ureteral catheter was placed at the end of the procedure at the surgeon’s discretion. We removed the urethral and ureteral catheters after 24–48 h, and the DJ after 7–14 days. We do not routinely perform pre-stenting before RIRS.

#### Percutaneous nephrolithotomy

The renal puncture was performed under ultrasonographic and fluoroscopic guidance. A nephrostomic sheath was placed after one-shot dilation. The size of tract was decided according to our previously published paper [20].

Fragments were removed using the vacuum cleaner effect, baskets, or aspiration. An 8 ch nephrostomy tube and/or DJ stent or a ureteral catheter were placed at the end of the procedure at the surgeon’s discretion.

In the first postoperative day, the urethral and ureteral catheters were removed and the nephrostomy tube was closed. An antegrade nephrogram was taken 24–28 h after the procedure. The tube was removed if no extravasation or retained calculi were present.

In all cases of RIRS and PCNL, we used a holmium:YAG laser fiber for lithotripsy and a non-pressurized irrigation system.

### Statistical analysis

Medians and interquartile ranges were reported for non-normally distributed continuous variables and frequencies for categorical variables. Sensitivity, specificity, positive predictive value (PPV), and negative predictive value (NPV) of urine cultures for detecting a positive SC or postoperative SIRS were calculated. The associations between the analyzed factors and SIRS and positive SC were evaluated with logistic regression. Statistical analyses were performed using IBM SPSS version 21 (SPSS, Chicago, IL). Significance was assumed at the 0.05 level.

## Results

### Baseline and perioperative characteristics

The study included 107 consecutive patients: 54 underwent RIRS and 53 PCNL.

Table 1 depicts clinical and demographic characteristics of patients, stone’s parameters, and the perioperative variables. During RIRS, an UAS was used in 51/54 procedures (94.4%). In 26/51 patients (50.9%), a ureteral stent was positioned before the RIRS in an urgent setting for acute decompression of the renal cavity.

**Table 1:** Peri-operative variables of patients and stone’s characteristics

Parameter	Overall (*n* = 107)	RIRS (*n* = 54)	PCNL (*n* = 53)
**Preoperative**			
Age at surgery (years)	55 (46–64)	55 (47.2–62.7)	54 (45–64)
Body mass index (kg/m^2^)	24.9 (22–28)	24.9 (22.3–29)	24.9 (22.8–27.9)
Male *n* (%)	70 (65.4%)	30 (55.6)	40 (75.5)
Female *n* (%)	37 (34.6%)	24 (44.4)	13 (24.5)
Charlson Comorbidity Index			
0	60 (56,1%)	30 (55.6)	30 (56.6)
1	16 (14.9%)	10 (18.5)	6 (11.3)
≥ 2	31 (29%)	14 (25.9)	17 (32.1)
Diabetes *n* (%)	16 (15%)	11 (20.4)	5 (9.4)
Left side *n* (%)	55 (51.4%)	27 (50)	28 (52.8)
Right side *n* (%)	52 (48.6%)	27 (50)	25 (47.2)
Maximum stone diameter (mm)	14 (10–23)	10 (8.25–14)	20 (15–30)
Hounsfield units value	1000 (600–1200)	1050 (546–1253)	1000 (620.5–1180)
Single stone *n* (%)	36 (33.6%)	23 (42.6)	13 (24.5)
Multiple stones *n* (%)	71 (66.4%)	31 (57.4)	40 (75.5)
Staghorn stones *n* (%)	43 (40.2%)	7 (12.9)	36 (67.9)
History of previous stone surgery *n* (%)^a^	70 (65.4%)	36 (66.7)	34 (64.1)
Pre-operative DJ stent *n* (%)	43 (40.2%)	26 (48.1)	17 (32.1)
Pre-operative nephrostomy tube *n* (%)	6 (5.6%)	1 (1.8)	5 (9.4)
History of previous urinary tract infection *n* (%)	35 (32.7%)	15 (27.8)	20 (37.7)
**Intraoperative**			
Operative time (minutes)^b^	120 (94–150)	102.5 (85–130)	138 (106–175)
Percutaneous tracts number			
1 *n* (%)	–	–	45 (84.9%)
≥ 2 *n* (%)			8 (15.1%)
PCNL type			
Mini-PCNL *n* (%)	–	–	34 (64.2%)
Regular-PCNL *n* (%)			19 (35.8%)
UAS diameter			
10–12 ch *n* (%)	–	15 (27.8)	–
11–13 ch *n* (%)		36 (66.7)	
Exit strategy (*n* = 54)			
Ureteral catheter *n* (%)	21 (19.6)	19 (35.2%)	2 (3.7%)
DJ stent *n* (%)	35 (32.7)	35 (64.8%)	0
Nephrostomy tube *n* (%)	30 (28.1)	0	30 (56.6%)
Nephrostomy tube + DJ stent *n* (%)	11 (10,3)	0	11 (20.8%)
Nephrostomy tube + ureteral catheter *n* (%)	10 (9.3)	0	10 (18.9%)
Intraoperative complications *n* (%)			
Yes	11 (10.3%)	1 (1.9)	10 (18.9)
No	96 (89.7%)	53 (98.1)	43 (81.1)
Intraoperative stone-free status *n* (%)			
Yes	83 (77.6%)	46 (85.2)	37 (69.8)
No	24 (22.4%)	8 (14.8)	16 (30.2)
Stone composition *n* (%)			
Calcium	63 (58.9%)	38 (70.5)	25 (47.2)
Infectious^c^	23 (21.5%)	8 (7.5)	15 (28.3)
Uric acid	20 (18.7%)	8 (7.5)	12 (22,6)
Cystine	1 (0.9%)	0	1 (1.9)
**Postoperative**			
Postoperative complications *n* (%)			
None	77 (72%)	46 (85.2%)	31 (58.5%)
Clavien–Dindo grade I	10 (9.3%)	5 (9.3%)	5 (9.4%)
Clavien–Dindo grade II	16 (15%)	3 (5.5%)	13 (24.5%)
Clavien–Dindo grade IIIa	3 (2.8%)	0	3 (5.7%)
Clavien–Dindo grade IIIb	1 (0.9%)	0	1 (1.9%)
Length of stay (days)	3 (2–6)	3 (2–3.25)	3 (3.5–7.5)
Readmission *n* (%)			
Yes	4 (3.7%)	1 (1.9)	3 (5.7)
No	103 (96.3%)	53 (98.1)	50 (94.3)

Medians and interquartile ranges (IQR) for continuous variables and frequencies for categorical variables

^a^Included: ureteral stent, nephrostomy tube, ESWL, URS-RIRS and PCNL

^b^Operative time is considered as the total duration of the procedure (from the preliminary cystoscopy to the final urethral catheter placement)

^c^Included: magnesium ammonium phosphate and carbonate apatite

### Cultures results

A positive SFUC, BUC, RPUC, or SC was found in 17 (15.9%), 22 (20.6%), 26 (24.3%), and 30 (28%) patients, respectively.

All 4 specimens were simultaneously positive in 10 patients (9.3%) and negative in 71 (66.3%). The concordance between SFUC and SC was the highest among all cultures: 94.1%, versus 86.4% for BUC and 84.6% for RPUC. SFUC and SC grew identical microorganisms in 15/17 (88.2%) patients with positive SFUC. Rather, BUC and SC had microbiological concordance in 12/22 (54.5%), and RPUC and SC in 17/26 (65.4%) patients.

Table 2 reports sensitivity, specificity, PPV, and NPV of BUC, RPCU, and SFUC to detect a positive SC.

**Table 2:** Predicting stone culture positivity and SIRS using various specimens

Sample	Sensitivity		Specificity		PPV		NPV	
	For positive SC	For SIRS	For positive SC	For SIRS	For positive SC	For SIRS	For positive SC	For SIRS
Bladder urine culture	63.3%	37.5%	96.1%	82.2%	86.4%	27.3%	86.9%	88.1%
Renal pelvic culture	73.3%	58.8%	94.8%	82.2%	84.6%	38.5%	90.1%	91.4%
Stone fragmentation urine culture	53.3%	41.2%	98.7%	88.9%	94.1%	41.2%	84.4%	88.9%
Stone culture	/	58.8%	/	77.8%	/	33.3%	/	90.9%

In SC and RPUC, the most frequent isolated pathogen was *E. faecalis*. In contrast, the bladder urine was predominantly infected by Gram-negative pathogens. In some cases, SFUC were positive for a mix of the pathogens identified in RPUC and SC (Fig. 1 Supplementary Material).

According to SC AST, the rate of multidrug resistant pathogens was 16.6%, all Gram negative. Among patients with a positive SC, the result of this culture prompted an antibiotic change in six patients (20.7%) with postoperative infectious complications.

### Infectious complications

Sixteen patients (15%) developed fever (> 38 °C). Out of 17 (15.9%) patients with SIRS, 8 (7.5%) had sepsis. Sepsis requiring intensive care or septic shock never occurred.

In two cases, the blood cultures were positive for ampicillin-sensitive *E. faecalis*: in one patient the SFUC and SC were positive for *E. faecalis*; in the other case the same pathogen was found in both the RPUC and SC. Among eight patients who developed sepsis, 6 (75%) revealed a positive SC and 3 (37.5%) a positive SFUC. 12/17 (70.6%) patients with a positive SFUC developed infectious complications, while only 34.6% of patients with positive RPUC had fever/SIRS or sepsis.

Table 2 reports the sensitivity, specificity, PPV, and NPV of the different cultures in predicting SIRS.

### Univariate analysis

Univariate logistic regression analysis revealed that previous urinary tract infections, a preoperative nephrostomy, stone diameter and composition, staghorn calculi, PCNL, and a positive BUC, RPUC, or SFUC were all predictors of a positive SC (Table 1 Supplementary Material).

Variables suggestive of a complex stone (stone diameter, Hounsfield Units, staghorn, and infectious stones), a complex PCNL (operative time, multiple tracts and blood transfusion), and an infection of the upper tract, but not of the bladder, were associated with the risk of postoperative SIRS (Table 2 Supplementary Material).

## Discussion

We found a high concordance between SFUC and SC, suggesting that the SFUC could be considered a novel tool to identify stone-colonizing bacteria and could potentially replace the need for a SC. SFUC had the highest PPV and specificity in detecting positive SC and SIRS.

In our study, the sensitivity and specificity of SC in predicting SIRS were lower than those reported by Mariappan (58.8% vs 73.7% and 77.8% vs 81.8%, respectively) [7]. However, in our series, SIRS and sepsis were significantly higher in patients with positive RPUC, SFUC, or SC but not in patients with a positive BUC. Our high discordance rate of 50% between the BUC and SC among patients with postoperative sepsis was similar to that reported by Nevo (47%) [21].

Our results demonstrate that, even when preoperative BUC is treated appropriately, the SC reveals the same pathogen in 14/22 patients (63.6%), implying that the bacteria are harbored inside the stone. These results confirm the findings from Patel et al., who found that 53% of postoperative cultures grew the same bacteria identified in the preoperative cultures [22].

The utility of SC would be to change the antibiotic therapy that would have been used based only on the BUC. The rate of antibiotic switching according to SC reported in the literature is very heterogeneous: from 1.3 to 64% [3, 21, 23, 24]. Our rate of antibiotic switching (20.7%) confirmed the clinical utility of SC. On the contrary, Osman et al. reported that SC was clinically useful only in 1 patient with positive SC [25]. Despite a similar rate of SC positivity (28% in our series vs 29.1%), we included more patients (107 vs 79) and a greater number of stone variables.

Our results confirm that pathogens colonizing stones are different from those isolated in bladder urine [3]. Our sample was prevalently Gram positive in the stone and upper tract urine compared to predominantly Gram negative in the bladder. Moreover, *E. faecalis* was the most common pathogen found in the upper tract cultures of patients who developed SIRS and sepsis (44.4% and 50%, respectively).

These findings are consistent with many studies that confirmed the shift of the microbiology of stone disease during the last generation from predominantly Gram-negative to predominantly Gram-positive organisms [3, 8, 21]. There are many possible explanations. Several groups have documented the decreasing frequency of struvite stones in the neurogenic patient population [26, 27]. Moreover, the major advances in the urological care of patients with structural and neurogenic disorders have had an impact on decreasing stones resulting from ureolysis (usually, urea-splitting bacteria are Gram negative). The developments in the minimally invasive treatment of urolithiasis may also be a factor in the microbiology of stone disease. The endoscopic treatment may entail repeated episodes of instrumentation of the urinary tract, providing opportunities for the introduction of Gram-positive organisms.

Similar to the study by Korets et al. [8], our univariate analysis confirmed that complex stones (staghorn, infected, large, hard) in infected urine that require a complex PCNL procedure (long, with multiple access) put the patient at risk for SIRS. Of note, in our study, female gender was not a risk factor for a positive SC or for SIRS. Accordingly, in our cohort, the rate of positive BUC and SC was equal in both females and males (50% in both cases). This may be partly explained by the low incidence of *P. mirabilis* infections in our cohort, historically associated with struvite stones more common in females [28].

Especially in the subset of patients at higher risk for SIRS, a systematic culture of the upper tract urine and stone is essential for indicating the most effective postoperative management. Moreover, in these cases, perioperative antibiotic management should cover Gram-positive bacteria.

We are the first, to our knowledge, to report the use of SFUC during RIRS and PCNL and its correlation with SC and SIRS, and to investigate the role of SC during RIRS.

A recent paper analyzed the spreading of bacteria in the irrigation fluid and blood during RIRS, showing that 26.3% of patients had a positive irrigation fluid sample [29]. The authors concluded that irrigation fluids cultures had no role in predicting infectious complications. Our rate of positive SFUC is lower, but a direct comparison appears difficult because in the study from Cai et al., more than one sample per patients was collected, probably enhancing sensitivity. Moreover, intraoperatively identified strains were not correlated with those isolated from postoperative urine and blood cultures, thus neglecting the role of irrigation fluids cultures in prompting postoperative antibiotic therapy variations. Additionally, a direct comparison with SC was not performed. In our series, out of 17 patients with a positive SFUC, 16 had a positive SC, strengthening the hypothesis that during fragmentation the pathogens are released from the calculi.

One limitation of this study is the lack of a multivariable analysis to adjust for potential confounders due to the small number of patients with infective complications.

Obviously, our results represent the local bacterial flora, which may limit the applicability to other centers. Also, we do not discuss economic considerations, due to the variability between different centers.

Our sepsis rate is not negligible, but probably reflects the complex population of our tertiary referral center and may be related to the adherence to the definition of sepsis as SIRS associated with suspicion or confirmation of a urinary pathogen.

It is known that SIRS may also be related to non-infectious causes and can be considered oversensitive and non-specific. However, we excluded other potential causes of fever, tachycardia/SIRS, such as atelectasis/pneumonia, hypovolemia, or uncontrolled pain.

Despite the SIRS concept being removed from The Third International Consensus Definitions for Sepsis and Septic Shock [30], we still deliberately employ it because fever alone cannot be used as an indicator for systemic infection, and sepsis is not common in the postoperative period. Moreover, the parameters used to define SIRS are easily measurable and reproducible in everyday urological clinical practice, in contrast to the Sequential Organ Failure Assessment parameters [27]. Thus, SIRS criteria may still be a useful screening tool for identifying patients with infection.

Another limitation is the heterogeneity of our population, which included PCNL and RIRS patients and consequently different stones and techniques. We decided to include both surgeries because one of our primary aims was to evaluate the feasibility of detecting bacterial growth in the irrigation fluid during stone fragmentation.

## Conclusions

In this study, we demonstrated that SFUC is technically feasible, easy to retrieve and to analyze.

We also confirmed the utility of the SC in the postoperative management of patients with infectious complications, including patients submitted to RIRS. In our opinion, considering the elevated microbiological concordance between SFUC and SC, SFUC could be useful in clinical practice during endourological procedures in which a stone fragment cannot be sent for SC, for example, in cases of small calculi (e.g., the only fragment retrieved is used for stone analysis), complete dusting, or during micro-PCNL. Larger studies will be needed to investigate the clinical utility and cost effectiveness of SFUC during stone surgery.

## Electronic supplementary material

Below is the link to the electronic supplementary material.

Supplementary file1 (DOCX 183 kb)
