# Supplementary material for: Feasibility and relevance of urine culture during stone fragmentation in patients undergoing percutaneous nephrolithotomy and retrograde intrarenal surgery: a prospective study
Source: World J Urol. 2020 Jul 30;39(6):1725–32. doi: 10.1007/s00345-020-03387-6 (PMC8217000; doi:10.1007/s00345-020-03387-6)
Supplement: Supplementary file 1 — Supplementary file1 (DOCX 183 kb) [file 345_2020_3387_MOESM1_ESM.docx]

**SUPPLEMENTARY MATERIAL**

Figure 1. Types of microorganisms cultured among different specimens: bladder urine culture (n= 22), renal pelvic urine culture (n= 26), stone fragmentation urine culture (n= 17), stone culture (n= 30)


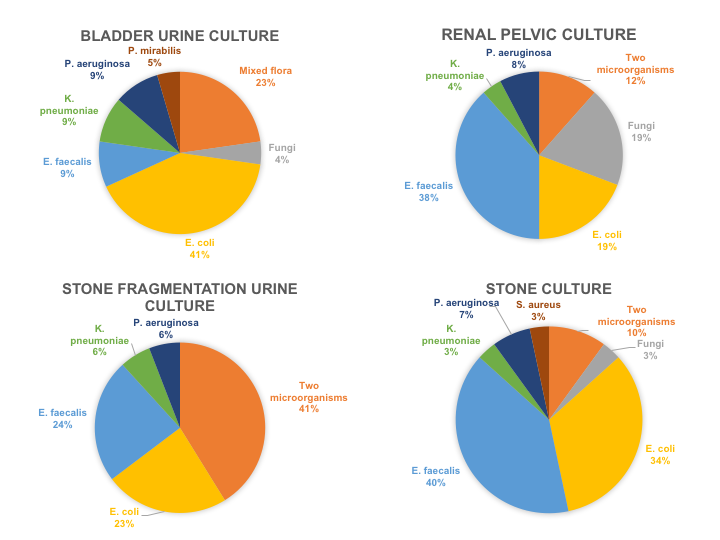


Table 1. Univariate analysis of risk factors for positive stone culture

| **VARIABLES** | **p-value** |
| --- | --- |
| *Patient related*  - age  - Body mass index  - Charlson Comorbidity Index  - gender  - diabetes  - previous surgery  - pre-operative nephrostomy tube  - pre-operative stent | 0,9  0,052  0,4  0,055  0,065  0,2  0,036  0,8 |
| *Stone related*  - maximum stone diameter  - Hounsfield Units  - multiple stones  - staghorn calculi  - stone composition (infectious stones) | 0,002  0,4  0,2  <0,001  0,03 |
| *Surgery related*  - surgery (PCNL)  - multiple tracts | 0,001  0,8 |
| *Microbiological variables*  - previous urinary tract infection  - positive bladder urine culture  - positive renal pelvic urine culture  - positive stone fragmentation urine culture | <0,001  <0,001  <0,001  <0,001 |

Table 2. Univariate analysis of risk factors for SIRS

| **VARIABLES** | **p-value** |
| --- | --- |
| *Patient related*  - age  - Body mass index  - Charlson Comorbidity Index  - gender  - diabetes  - previous surgery  - pre-operative nephrostomy tube  - pre-operative stent | 0,08  0,85  0,59  0,946  0,059  0,534  0,229  0,712 |
| *Stone related*  - maximum stone diameter  - Hounsfield Units  - multiple stones  - staghorn calculi  - stone composition (infectious stones) | 0,004  0,027  0,336  0,03  0,03 |
| *Surgery related*  - surgery (PCNL)  - operative time  - intraoperative stone-free status  - multiple tracts  - blood transfusion | 0,001  0,015  0,05  0,02  0,015 |
| *Microbiological variables*  - previous urinary tract infection  - positive bladder urine culture  - positive renal pelvic urine culture  - positive stone fragmentation urine culture  - positive stone culture  - multi-drug resistant bacteria on stone culture | 0,053  0,08  0,001  0,004  0,004  0,4 |
